# Enzymatic Activity and Horizontal Gene Transfer of Heavy Metals and Antibiotic Resistant *Proteus vulgaris* from Hospital Wastewater: An Insight

**DOI:** 10.1155/2022/3399137

**Published:** 2022-12-06

**Authors:** Manzar Alam, Nilofer Bano, Tarun Kumar Upadhyay, Reem Binsuwaidan, Nawaf Alshammari, Amit Baran Sharangi, Radhey Shyam Kaushal, Mohd Saeed

**Affiliations:** ^1^Department of Biosciences, Integral University, Lucknow 226026, Uttar Pradesh, India; ^2^Department of Bioengineering, Integral University, Lucknow 226026, Uttar Pradesh, India; ^3^Department of Biotechnology, Parul Institute of Applied Sciences and Centre of Research for Development, Parul University, Vadodara 391760, Gujarat, India; ^4^Department of Pharmaceutical Sciences, College of Pharmacy, Princess Nourah Bint Abdulrahman University, Riyadh, Saudi Arabia; ^5^Department of Biology, College of Science, University of Ha'il, Ha'il, P.O. Box 2440, Saudi Arabia; ^6^Department of Plantation Spices Medicinal and Aromatic Crops, Bidhan Chandra Krishi Viswavidyalaya, Mohanpur 741252, West Bengal, India

## Abstract

Globally, the issue of microbial resistance to medicines and heavy metals is getting worse. There are few reports or data available for *Proteus vulgaris* (*P. vulgaris*), particularly in India. This investigation intends to reveal the bacteria's ability to transmit genes and their level of resistance as well. The wastewater samples were taken from several hospitals in Lucknow City, India, and examined for the presence of Gram-negative bacteria that were resistant to antibiotics and heavy metals. The microbial population count in different hospital wastewaters decreases with increasing concentrations of metal and antibiotics. Among all the examined metals, Ni and Zn had the highest viable counts, whereas Hg, Cd, and Co had the lowest viable counts. Penicillin, ampicillin, and amoxicillin, among the antibiotics, demonstrated higher viable counts, whereas tetracycline and erythromycin exhibited lower viable counts. The MIC values for the *P. vulgaris* isolates tested ranged from 50 to 16,00 *μ*g/ml for each metal tested. The multiple metal resistance (MMR) index, which ranged from 0.04 to 0.50, showed diverse heavy metal resistance patterns in all *P. vulgaris* isolates (in the case of 2–7 metals in various combinations). All of the tested isolates had methicillin resistance, whereas the least number of isolates had ofloxacin, gentamycin, or neomycin resistance. The *P. vulgaris* isolates displayed multidrug resistance patterns (2–12 drugs) in various antibiotic combinations. The MAR indexes were shown to be between (0.02–0.7). From the total isolates, 98%, 84%, and 80% had urease, gelatinase, and amylase activity, whereas 68% and 56% displayed protease and beta-lactamase activity. Plasmids were present in all the selected resistant isolates and varied in size from 42.5 to 57.0 kb and molecular weight from 27.2 to 37.0 MD. The transmission of the antibiotic/metal resistance genes was evaluated between a total of 7 pairs of isolates. A higher transfer frequency (4.4 × 10^−1^) was observed among antibiotics, although a lower transfer frequency (1.0 × 10^−2^) was observed against metals in both the media from the entire site tested. According to exponential decay, the population of hospital wastewater declined in the following order across all sites: Site II > Site IV > Site III > Site I for antibiotics and site IV > site II > site I >site III for metal. Different metal and antibiotic concentrations have varying effects on the population. The metal-tolerant *P. vulgaris* from hospital wastewater was studied in the current study had multiple distinct patterns of antibiotic resistance. It could provide cutting-edge methods for treating infectious diseases, which are essential for managing and assessing the risks associated with hospital wastewater, especially in the case of *P. vulgaris*.

## 1. Introduction

Heavy metal contamination is becoming more widespread due to its heavy use in industrial processing. Heavy metals-laden industrial wastewater causes health risks to human, animal, and aquatic life [1]. Lead and cadmium, the critical contaminants found on the Earth, are extremely harmful to humans, animals, plants, and microorganisms. Bacterial resistance and metal tolerance capabilities are typical phenomena that might be used for environmental bioremediation. Hence, these resistant bacteria could be one of the most viable biotechnological options. However, the hazards posed by antibiotic-resistant and heavy-metal-tolerant bacteria can be exploited in detoxification processes to convert a hazardous form of drug to a safe form by establishing biotransformation mechanisms [2]. Antibiotic resistance can quickly spread among *P. vulgaris*. The effectiveness of antibiotics in the treatment of infectious diseases is known to be harmed by the presence of antibiotic-resistant bacteria in natural habitats. If microorganisms are becoming highly resistant to antibiotics, humankind is continually pushed to invent new antibiotic derivatives [3]. Antimicrobial resistance is one of the most serious issues of the present time, which can cause long-term threats if it remains untreated. As a result, it is necessary to understand bacterial resistance properties and create regional antibiotic resistance profiles in natural regions [3]. In microbiological ecosystems, aquatic settings provide a means of spreading antibiotic-resistant bacteria and resistant genes [4]. Microbes can develop resistance against antibiotics by producing extracellular enzymes that destroy or deactivates the antibiotics. Cephalosporins, penicillins, monobactams, and carbapenems, for example, are classified chemically as beta-lactam antibiotics, and many bacteria develop resistance to these antibiotics by developing different beta-lactamases that can neutralize some forms of these antimicrobials. Beta-lactamases are enzymes that break the beta-lactam ring of antibiotics, destroying them [5]. The production of extended-spectrumbeta-lactamases is a remarkable sensitivity method that inhibits the antibacterial treatment of infections caused by Gram-negative bacteria and is a major issue for present antimicrobial drugs [6]. *Proteus* spp. is Gram-negative bacilli having a place with the family *Enterobacteriaceae*. Bacteria belonging to the *Proteus* spp. are generally known to be opportunistic in causing human infections. Their involvement in human disease, as well as their virulence factors, allow bacteria to enter diverse niches of the host organism [4]. *Proteus* spp. is believed to be a major concern with the widely occurring infections in hospitals as community-acquired diseases [4, 7]. This pathogen features a different strategy for spread and thus can cause contamination in several anatomical goals of the body. A bit of the ensnaring sources of transmission is soil, contaminated water, sustenance, supplies, intravenous plans, patient's hands, and hospital staff [8]. *Proteus* spp. infects patients who have a weakened immune system, and almost all of them can cause urinary tract and wound infections, as well as nosocomial infections. Urinary tract infections are most commonly emerging and are typically linked to the use of urinary catheters. It is worth noting that *Proteus* spp. is the most common bacteria found in bladder and kidney stones [9, 10]. Plasmids usually do not carry genes that are required for the host's survival in “nonstress” conditions, and their origin of replication (ori) regulates the number of copies they make. Since two plasmids containing the same replication origin cannot coexist in the same bacterium and are thus incompatible, the variety of the ori genes is usually working for their classification [11]. Integrons are not self-transferable structures; they are most often located on plasmids or transposons, allowing their efficient horizontal gene transfer [12]. Integrative conjugative components are mobile genetic elements that can be excised from the chromosome by the host bacterium's integrase/excisionase gene, forming a circular intermediate that can be transferred by conjugation [13]. Sulfamethoxazole, trimethoprim, chloramphenicol, Mercury, streptomycin, and kanamycin resistance genes, as well as beta-lactamases genes (blaHMS-1, blaCMY-2, and blaCTX-M-2) are all present [14]. Bacteria are becoming more resistant to antibiotics as a result of the overuse of drugs and inappropriate handling of hospital wastewater that contains heavy metals, hazardous substances, and radioactive materials. Heavy metal plays a significant part in spreading antibiotic resistance in the microorganisms from the environment. Numerous studies have focused on the relationship between antibiotic and heavy metal resistance, and the same plasmid expressed both antibiotic and heavy metal resistance. Antibiotic resistance among *P. vulgaris* strains that can tolerate heavy metals has been the subject of recent research. The present work also examined plasmid size and horizontal gene transfer frequency among selected *P. vulgaris* isolates. Several unique patterns of antibiotic resistance were analysed among the metal-tolerant *P. vulgaris* isolates. All the medications used in this experiment were further validated using Lipinski's rule of five and ADME-Tox were discovered to fit within the range of drug-like properties. This opens the door for further investigation into the prevalence of antibiotic resistance in *P. vulgaris* infections found in hospital settings.

## 2. Methodology

### 2.1. Sample Collection

Three hospitals in Lucknow, Uttar Pradesh, India, were each assigned a site number for their waste effluents, including King George's Medical University site (I), Sanjay Gandhi Post Graduate Institute of Medical Sciences (treated) site (II), Sanjay Gandhi Post Graduate Institute of Medical Sciences (untreated) site (III), and Dr. Ram Manohar Lohia Hospital site (IV). Test samples were collected in sterile 250 ml polypropylene bottles in accordance with the generally accepted procedure [15]. Test samples were kept at 4°C until they reached the lab.

### 2.2. Isolation and Identification of Metal-TolerantGram-Negative Bacteria

Tests for the isolation of metal-resistant Gram-negative bacteria from wastewater were carried out at various concentrations (ranging from 25 to 1200 g/ml) on metal-added MacConkey agar plates. On MacConkey agar medium, Gram-negative bacterial counts, comprising pink and colourless colonies, were measured as CFU/ml after plates were incubated for 24 hours at 37°C. The final step was to identify these isolates using their morphological, cultural, and biochemical traits [16].

### 2.3. MIC of Heavy Metals among *P. vulgaris* Isolates

To assess the level of heavy metal resistance, the MIC against *P. vulgaris* isolates was utilised [17]. Additional plates of each heavy metal were created, with 100 g/ml to 2000 g/ml concentrations. Using a platinum circle with a diameter of 5 mm, inoculums of the test strain (3 × 10^6^ CFU/ml) were applied in duplicate to heavy metal modified plates and control plates. In order to observe *P. vulgaris* isolate growth on the inoculated spotted plates, the plates were maintained at 37°C for 24 hours. The lowest inhibitory concentration (MIC) of the heavy metal that inhibits the test isolates from growing visibly was used to define the MIC.

### 2.4. Multiple Metal Resistance (MMR) Indexing

The risk to environmental health was evaluated using the MMR index profile based on the isolate and sample site. MMR index for test isolates was determined in accordance with the following equation (1):

(1) $$\begin{array}{c} MMR\;index=\frac{No.\;of\;metal\;to\;which\;all\;isolates\;were\;resistant}{No.\;of\;metal\;tested\;X\;No.\;of\;isolates}\text{.} \end{array}$$

### 2.5. Isolation of Antibiotic-ResistantGram-Negative Bacteria

On antimicrobial-amended MacConkey agar plates, Gram-negative microbes that are resistant to antibiotics were isolated from hospital wastewater at varying concentrations (10–160 g/ml). To assess the overall population of antibiotic-tolerant Gram-negative bacteria, 0.1 ml of wastewater was spread on MacConkey agar medium and plated in serial dilutions. On MacConkey agar medium, Gram-negative bacterial counts were expressed as CFU/ml, and plates were incubated at 37°C for 24 hours.

### 2.6. Determination of MIC of Antibiotics among *P.vulgaris* Isolates

The MIC of five different antibiotics was measured by using the plate dilution method (penicillin, erythromycin, tetracycline, amoxicillin, and ampicillin). The antimicrobials were added to nutrient agar at various concentrations ranging from 5 to 640 g/ml, increased separately, and then spot-inoculated with about 3 × 10^6^ microbial cells by using a platinum loop with a diameter of 5 mm. Additionally, the plates were incubated for 24 hours at 37°C. The MIC was defined as the lowest concentration of antibiotics that prevented microbial growth.

### 2.7. Antibiotic Sensitivity Test

CLSI [18] advised that the common disc diffusion technique be used to measure antibiotic resistance by using Mueller-Hinton agar (Difco) and *E. coli* ATCC 25922 as a control strain. Nalidixic acid (NA) 30 g, amoxicillin (AMX) 25 g, gentamycin (GEN) 30 g, neomycin (N) 30 g, nitrofurazone (NR) 100 g, ampicillin (AMP) 10 g, chloramphenicol (CHL) 30 g, kanamycin (K) 30 g, polymixin B (PB) 300 g, methicillin (M) 5. After the discs were applied, the plates were turned over and maintained at 37°C for an additional 15 minutes. The plates were checked, and the diameters of the whole inhibition zones were measured after 24 hours of incubation. Zone diameter classifications for both sensitive and resistant antimicrobial agents were then given.

### 2.8. Multiple Antibiotic Resistances (MARs) Indexing

To assess the environmental health risk, the MAR index profile based on the isolate and sampling site was performed. Equation (2) [19] was used to determine the MAR record for the test strain.

(2) $$\begin{array}{c} MAR\;index=\frac{No.\;of\;antibiotic\;to\;which\;all\;isolates\;were\;resistant}{No.\;of\;antibiotics\;tested\;X\;No.\;of\;isolates}\text{.} \end{array}$$

### 2.9. Extracellular Enzymatic Activities

#### 2.9.1. Beta-Lactamase Production

A circle loaded with developed culture was transferred into an Eppendorf tube containing 1 ml of penicillin G solution and incubated at 37°C for 30 minutes to recognize any microscopic organisms that produced beta-lactamases. 0.5 ml of iodine solution was added, and it was mixed for two to three minutes. Color shifts that become colorless indicate a successful outcome [20].

#### 2.9.2. Amylase Test

Isolates of *P. vulgaris* were streaked on starch agar medium plates and incubated for 24 to 48 hours at 37°C. After incubation, the plates were flooded with iodine solution by using a dropper. The iodine solution was then removed from the plates after being left undisturbed for 5–10 minutes. [16]. The development of a light yellow zone around a colony in a blue medium indicated the presence of starch-degrading activity. The isolates were deemed capable of producing amylase.

#### 2.9.3. Protease Test

The *P. vulgaris* isolate's protease-producing enzymatic activity was examined, and the test organisms were inoculated in a single streak onto the skimmed milk agar surface. For 24–48 hours, the plates were incubated inverted at 37°C. A distinct zone was seen surrounding the line of growth, which suggested a successful outcome.

#### 2.9.4. Lipase Test

*P. vulgaris* isolates were inoculated on a tributyrin agar medium. After 7 days of incubation at 28°C, a distinct halo zone appeared surrounding the colony, signalling the presence of lipase activity [16].

#### 2.9.5. Catalase Test

Trypticase soy agar medium slants were made, and the cultures' ability to produce catalase was tested. Slants were inoculated with the test microbe and kept at 37°C for 24–48 hours. Then, 3-4 drops of H_2_O_2_ gas were poured; the release of gas bubbles indicated a successful outcome.

#### 2.9.6. Gelatinase Test

In the nutrient gelatin plate technique, there is a test to determine gelatin hydrolysis. Currently, test microorganisms that are 18 to 24 hours old are stab inoculated onto culture plates that have already been loaded with nutrient gelatin. Gelatin-seeded plates with nutrient inoculations are incubated for 24 hours at 35°C. Clear zones surrounding colonies that are gelatinous-positive are evidence of gelatin hydrolysis.

#### 2.9.7. Urease Test

To sterilize the urease agar, it was autoclaved at 15 lbs weight (121°C) for 15 minutes before being chilled to 50°C. At that moment, 1.0 g of glucose and 0.2% of phenol red were added. After being added, 6.0 ml of the molten base underwent an hour-long steam treatment before being cooled to 50°C. The slants/broth was examined for urease (red color) and nonurease by looking at the developed color (yellow color).

### 2.10. Molecular Isolation and Characterization of Plasmids

Small-scale plasmid DNA plans of multidrug-resistant *P. vulgaris* isolates were completed using the Birnboin and Doly [21] approach of alkaline lysis. 3.0 ml of Luria-Bertani broth was used to incubate a single bacterial colony for the duration of the night at 37°C and 220 rpm. The cell suspension was transferred to a 1.5 ml microcentrifuge tube and centrifuged at 10,000 rpm for two minutes (Eppendorf, Model 5415C). Resuspending the obtained cell pellet in 150 l of glucose-EDTA-Tris (GET) buffer (pH 8.0) required the use of vortexes to guarantee adequate mixing. In addition, 175 l of 0.4 N NaOH and 2% SDS were mixed with the cell suspension. The cylinder was thoroughly mixed before being cooled to −20°C for an additional 10 minutes. After being completely mixed, 250 l of cool 5 M potassium acidic acid was added, and the mixture was left to sit at room temperature for 10 minutes. The tube was then centrifuged again for a further five minutes at a speed of 12,000 rpm. The supernatant was then transferred to a fresh 1.5 ml centrifuge tube, and cold isopropanol was then added. Gently combining the mixture, it was centrifuged once more for ten minutes at 12,000 rpm, and the DNA pellet was then washed with 650 ml of cooled 70% ethanol. The pellet was dried for 30 minutes and then resuspended in 40 ml of sterile deionized water after the supernatant was discarded. A minute-long staining procedure using ethidium bromide (0.5 g/ml) solution was performed after the contained plasmid had been electrophoresed on 0.8% agarose gel for an hour at 90 volts. The gel was then shown by using a gel documentation system. The molecular weight of the plasmid DNA of the multidrug-resistant* P. vulgaris* isolates was calculated by using the graphical technique connecting the logarithm of the subatomic load of *E. coli* V517 plasmids [22].

### 2.11. Resistance Transfer (Conjugation)

The MDR strain of *P. vulgaris* isolates exhibiting antibiotic and metal resistance were picked for horizontal gene transfer studies, which served as donors, while *P. vulgaris* isolates showed a large effect against antimicrobial agents under assessment and were used as a recipient. Conjugation was performed as similarly portrayed elsewhere [23]. All beneficial Gram-negative bacterial isolates and donor *P. vulgaris* isolates were quickly grown with antibiotic supplemented till the O.D. of each sort accomplished 0.85 at A600 (about 10^8^ cells/ml). As effluent, 2.5 ml of warm supplement mixture with 0.2 ml of each of the donor and recipient culture stocks were added. The combination was then incubated at 37°C without shaking. *P. vulgaris* isolates were then plated onto nutritional agar enriched with at least one of the antimicrobials/metals after 24 hours of incubation. The following antibiotics were used: streptomycin (S) 25 g, nalidixic destructive (NA) 30 g, neomycin (N) 30 g, cephradine (CH) 25 g, rifampicin (Rif) 2 g, gentamycin (GEN) 30 g, chloramphenicol (C) 30 g, penicillin (P) 10 g, and erythromycin (ERY) 15 *μ*g/Ni^2+^, Cd^2+^and Cr^6+^). It was demonstrated through the studies that concentrations of the above antimicrobial agents/heavy metals lead to problems in the development of a valuable strain of *P. vulgaris*. The transconjugants were recognized by their noticeable development on these counter-antibiotic-containing mediums. Controls (for donor and beneficiary strain) were continued running in each conjugation test concurrently, including comparable conjugation technique, but without including recipient culture stock (for donor control) and donor culture mixture. This was carried out to ensure that the transconjugants assurance was only provided by conjugation, not by change that could modify the counter microbial and metal vulnerability in the donor strains (for beneficiary control). Finding no development from controls and discernible development from the conjugation-mixed bacteria on nutrient agar/wastewater boosted with one of the aforementioned anti-infection agents revealed successful conjugation. The antibiotics of the donor and transconjugants were analysed by disc diffusion technique and MIC testing as described above. Transfer frequency was calculated as given in the following equation:

(3) $$\begin{array}{c} Frequency\;of\;transfer=\frac{CFU/ml\;of\;transconjugants}{CFU/ml\;of\;recipient}\text{.} \end{array}$$

### 2.12. Statistical Analysis

Data on cell counts at various metal concentrations were modified to fit the exponential decay model as follows:

(4) $$\begin{array}{c} N_{c}=N_{0}e^{{-}^{\beta }t}\text{,} \end{array}$$

where *N*_*c*_ is the number of bacteria at concentration *c*, *N*_0_ is the number of bacteria at zero concentration, and *c* is the metal concentration used.

Curve fitting was used to determine the slope (*β*), correlation coefficient (*R*), and residual mean square (RMS) values. The best bacterial cell count decay is represented by a higher *R*-value and a lower RMS value.

## 3. Results

In the current investigation, seven metals (Cr^6+^, Ni^2+^, Zn^2+^, Cu^2+^, Co^2+^, Hg^2+^, and Cd^2+^) at various concentrations (25 to 1200 g/ml) were tested against the heavy metal-tolerant community of Gram-negative bacteria from hospital wastewater samples. Compared with the corresponding cfu/ml of water in sites I, II, and III, Gram-negative bacteria demonstrated reduced metal-resistant tolerance viable counts in the range of 5.1 × 10^3^–1.0 × 10^2^ at 50–100 g/ml against every metal tested. Among all the examined metals, Ni and Zn had the highest viable counts, whereas Hg, Cd, and Co had the lowest viable counts. At various metal and antibiotic concentrations, all bacterial counts were fitted to an exponential decay model. Curve fitting was used to calculate *R* and RMS values for wastewater samples collected from various locations. Sites IV and II had the highest *R* values and the lowest RMS values at various metal and antibiotic concentrations. This demonstrates that the exponential decay model best fits the metal and antibiotic isolates from sites IV and II. The population of hospital wastewater were declined in the following order across all sites, according to exponential decay: for antibiotics, SiteII > Site IV > Site III > Site I, and for metal, site IV > site II > site I  >SiteIII. The effect of different metal and antibiotic concentrations on the population was variable (Tables 1 and 2).

**Figure 1:**
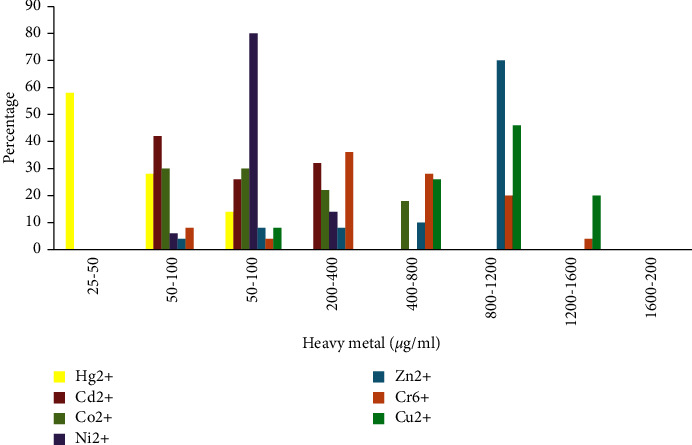
MIC range of heavy metals in *P. vulgaris* (*n* = 50) from entire sampling sites of hospital wastewater.

All the *P. vulgaris* isolates were tested for the MIC at different concentrations (25–1600 *μ*g/ml) of heavy metals (Hg^2+^, Cd^2+^, Cu^2+^, Zn^2+^, Ni^2+^, Co^2+^and Cr^6+^). The Hg^2+^ showed the highest toxicity against all the *P. vulgaris* isolates tested. 58% of the total isolates showed their MIC at the range of 25–50 *μ*g/ml for Hg^2+^, followed by 42% and 30% against Cd^2+^ and Co^2+,^ respectively. The MIC was not detected at the range of 25–50 *μ*g/ml against Cu^2+^, while at the range of 200–1600 *μ*g/ml against Hg^2+^, Cd^2+^, and Ni^2+^, respectively. 70% and 46% of the isolates showed their MIC at the range of 400–800 *μ*g/ml against Zn^2+^ and Cu^2+^(Figure 1).

Most *P. vulgaris* isolates from all the sites displayed resistance to two to seven metals simultaneously, in the same or different combinations. Maximum 40% and 24% of the isolates, respectively, displayed 6, 5, 4, and 3 distinct metal resistance patterns simultaneously in 3 and 5 different combinations. The *P. vulgaris* isolates from the hospital wastewater were found to have low- and high-risk MMR. All of the examined isolates had MMR indices that varied between 0.04 and 0.50 (Table 3).

**Figure 2:**
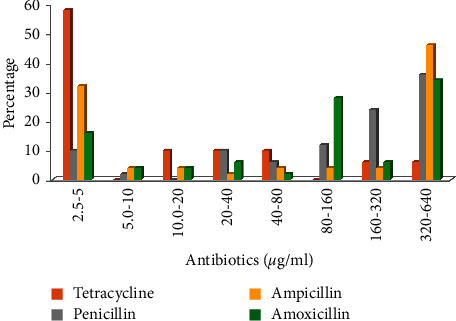
MIC range of antibiotics in *P. vulgaris* (*n* = 50) from entire sampling sites of hospital wastewater.

Gram-negative bacteria displayed increased antibiotic resistance, with a viable count range of 8.2 × 10^3^–4.0 × 10^2^ at 50–100 g/ml in site II, compared with 6.30 × 10^4^–5.0 × 10^2^, 5.0 × 10^4^–2.0 × 10^2^, and 3.60 × 10^4^–1.0 × 10^2^ cfu/ml of water in sites I, III, and IV, respectively. Additionally, the *P. vulgaris* resistance hierarchy was seen at various antibiotic concentrations. The MIC against five antibiotics was also determined for the total 50 *P. vulgaris* isolates. Tetracycline exhibited the highest level of toxicity against all isolates from all test sites. Maximum 58% of the isolates revealed their MIC for tetracycline in the 2.5–5 g/ml range, followed by 32%, 16%, 10%, and 2%, respectively, for ampicillin, amoxicillin, penicillin, and erythromycin. When ampicillin, penicillin, or amoxicillin were used at a concentration of 320–640 g/ml, 46%, 36%, and 34% of the isolates, respectively, showed their MIC (Figure 2).

Antibiotic resistance is also determined among *P. vulgaris* isolates. A high level of resistance was observed (100%) against methicillin, followed by 86%, 78%, 60%, 46%, and 38% to penicillin, ampicillin amoxicillin, polymixin, and nalidixic acid, while only 10%, and 6% of isolates, showed resistance against, nitrofurazone, gentamycin neomycin and ofloxacin, respectively (Table 4).

All of the isolates showed resistance to two, three, four, five, six, seven, nine, eleven, and twelve antibiotics simultaneously in the same or different combinations. Maximum 18% and 16% of all isolates, respectively, were found to be resistant to four, six, or seven antibiotics at once in four, seven, or six distinct combinations. The multiple antibiotics resistance (MAR) index was used to assess the isolates' potential for resistance. The MAR Index showed a diverse pattern among the isolates. All the examined isolates had MAR indices that ranged from 0.02–0.7 (Table 5).

Amylase, beta-lactamase, protease, lipase, gelatinase, and urease enzymatic activity were present in the majority of *P. vulgaris* isolates. Of the entire *P. vulgaris* isolates, 98%, 84%, 80%, 74, 68, and 56% showed activity for urease, gelatinase, amylase, lipase, catalase, protease, and beta-lactamase (Table 6).

The occurrence of plasmids in metal and multiple drug-resistant *P. vulgaris* was observed. All the multidrug and metal-resistant *P. vulgaris* isolates were found to contain a plasmid with different numbers, sizes, and molecular weights. The plasmids in the *P. vulgaris* isolates ranged in size from 42.5 to 57.0 kb and in molecular weight from 27.2 to 37.0 MD. 7 pairs of isolates were examined for the transfer of the antibiotic/metal resistance markers to determine the prevalence of resistance transfer. Mating sets showed a two-path exchange of resistance markers under nutrient broth and hospital wastewater. Single or different resistant markers were exchanged to the beneficiary isolates, which were sensitive to the markers. A high number of donors of *P. vulgaris* isolate demonstrated anti-infection metal resistance move in beneficiary *P. vulgaris* isolates and *E. coli* K-12 in supplement broth as compared with wastewater. Among all isolates, pairs of PR7-PR8, PR8-PR9, PR27-PR 46, PR30-PR4, PR38-PR17, PR43-PR-38, and PR47-PR25 showed a maximum of 3.4 × 10^–1^ and 3.1 × 10^–1^ frequency of resistance transfer among metal and antibiotic markers in a nutrient medium, while the pairs of PR8-PR9 and PR38-PR17 demonstrated maximum transfer frequency 4.4 × 10^−1^ and 3.1 × 10^−1^ among antibiotic and metal marker in wastewater from all the isolates tested, respectively. In the case of metals, the transfer frequency was observed at 3.3 × 10^–1^ and 1.1 × 10^−1^ for the isolates PR 17 in the nutrient medium and wastewater with resistance markers Ni^2+^, Cr^6+^ to the recipient *E. coli* K-12, while in the case of antibiotic, the transfer frequency 2.9 × 10^–1^ was observed by isolate PR17 in nutrient medium with marker AMX, AMP to the recipient *E. coli* k-12 (Tables 7–9).

### 3.1. Molecular Properties and Drug Likeness

Molsoft L.L.C. (https://molsoft.com/mprop/), which has the ability to compute the molecular properties with the use of valid structures and depends on these molecular properties, was used to explore the molecular properties and drug similarity of the chosen medications.

Molsoft L.L.C.'s drug-likeness and molecular property prediction tool and results are presented in Table 10 for all of the common medications that were used in this investigation.

### 3.2. ADMET/Tox Screening

Precisely, it integrates a system for the absorption, distribution, metabolism, and excretion properties of drug molecules by measuring various physicochemical properties. All the predicted ADME/Tox values are summarized in Table 11.

## 4. Discussion

Many bacteria that live in environments with harmful substances commonly exhibit heavy metal resistance. Over the past few years, metal resistance has advanced our understanding of the biological mechanisms involved. In a number of bacterial species, Mercury resistance has been made clear [24–26]. The occurrence or inadequacy of the heavy metal in the ground, specifically the decline in the Earth's capacity for tolerating heavy metals, breaks down the resistance of microscopic organisms to heavy metals [27]. Research studies [28] discovered that despite heavy metals enhancing resistance in the past, the discovery of tolerant microorganisms in environments with lower concentrations of heavy metals highlights the fact that heavy metal tolerance species are still alive today in unpolluted natural environments. The presence of microscopic organisms that can withstand metals in these circumstances may indicate that the area is impacted by heavy metals. By measuring the metal's MIC against *P. vulgaris*, we were able to assess the metal's resistance in our discovery. All of the *P. vulgaris* isolates tested showed that the Hg^2+^ was more hazardous. The bulk of the aggregated isolates (58%), followed by 42% and 30% for Cd^2+^ and Co^2+^, respectively, revealed that their MIC range for Hg^2+^ was 25–50 g/ml. In contrast to the range of 200–1600 g/ml against Hg^2+^, Cd^2+^, and Ni^2+^ individually, the MIC was not detected at 25–50 g/ml against Cu^2+^. This is consistent with several studies in which the author found that multidrug-resistance bacteria had higher MIC values than sensitive ones [29, 30]. The occurrence of metal resistance in this investigation is essentially consistent with those found elsewhere [26, 31, 32]. According to Malik and Aleem [33], the majority of bacterial isolates from heavy metal-contaminated soil testing displayed resistance to various metal ions. We also calculated the multiple metal resistance (MMR) index among *P. vulgaris* isolates across all sampling locations. Based on sample sites, isolates showed heterogeneity in their MMR index. *P. vulgaris* from the hospital wastewater was found to have low- and high-risk MMR. The MMR index of the *P. vulgaris* from the complete locations evaluated ranged from 0.04 to 0.5. Our findings were also reflected in how the other personnel had set things up. The majority of the isolates were found to be resistant to lead (94%) followed by nickel (40%), arsenate (35%), and copper (22%), according to Sabry et al. [34] the isolation of heterotrophic aerobic consuming heavy metal-tolerant bacterial community from harsh water. Ecological pollution is one of the greatest annoyances of the modern day, specifically contaminating the water in India due to the overuse of antimicrobials and other toxins. Studies have shown that the discharge of hospital wastewater increases the occurrence of antibiotic resistance [35]. The widespread use of antibiotics by humans is one factor contributing to the problem of antimicrobial resistance in microscopic organisms, which poses a real risk to civilization today [36, 37]. Antimicrobial resistance is alarmingly rising in microscopic organisms that cause either community infections or contaminations brought in by healthcare facilities. Antimicrobial resistance is the decline in a drug's efficacy in treating a disease or condition, such as an infection or a tumour. Antibiotic tolerance or dosage disappointment is equivalent terms because antibiotics are not intended to kill or stop a pathogen at that time. Multidrug resistance is the term used to describe an organism that is resistant to multiple antibiotics [38]. Many anti-infection drugs have chemical linkages, such as amides and esters, which are sensitive to hydrolysis. The antitoxin movement is known to be destroyed by a few substances that concentrate on and break these connections. These catalysts might discharge regularly. All penicillin, third-generation cephalosporins, and aztreonam are protected from bacterial growth by expanded range beta-lactamases (ESBLs), but cephamycins and carbapenems are not [39]. Increased use of antimicrobial drugs in the environment through restorative treatment, commercial agriculture, and organic farming has had a specific impact on the bacterial population [40]. Compared to isolates from human faeces, those from hospital wastewater were more resistant to antimicrobials. In this analysis of 15 anti-infection agents from the four testing locations, an abnormal level of antibiotic resistance was observed. The resistance of each isolate was examined for both single and multiple antibiotic resistances. All isolates tested positive for several antimicrobial agent resistances. Methicillin resistance was present in all isolates at 100%, while penicillin resistance was present in 86% of the isolates. In 38% of the isolates, Nalidixic acid resistance was present. All of the isolates tested positive for high sensitivity to the antibiotics chloramphenicol, ciprofloxacin, gentamycin, neomycin, and ofloxacin. Similar to this, all of the isolates were highly toxic to nitrofurazone, tetracycline, and erythromycin. The *Proteus* spp. was discovered to have strong antimicrobial resistance to tetracycline (85%), chloramphenicol (82.5%), co-trimoxazole (81%), and ampicillin (77%), according to Feglo et al. [41]. Comparative results from earlier in Ghana were revealed by Newman et al. [42]. Since unpredictable consumption of antimicrobial medications exerts specific pressure and leads to a higher prevalence of resistant microscopic organisms, the high antibiotic resistance of *Proteus* spp. may be an indication of resistance levels among Enterobacteriaceae and maybe *Salmonella* [43]. As part of our analysis, we also determined the level of antibiotic resistance for the *P. vulgaris* isolates to tetracycline, penicillin, amoxicillin, ampicillin, and erythromycin. All of the isolates showed that their MIC ranged between 5 and 640 g/ml for all of the tested antibiotics. The majority of isolates displayed their MIC at a lower level (5–10 g/ml) against tetracycline, whereas a lower level (10–20 g/ml) was reported against penicillin, and the majority of isolates displayed their MIC at 320–640 g/ml. The majority of isolates displayed MICs against erythromycin in the range of 5–320 g/ml. Most of the isolates had MIC values for amoxicillin and ampicillin of 320–640 g/ml and 640–800 g/ml, respectively. Numerous different investigations have taken into account comparative patterns of the drug MIC levels in members of the *Enterobacteriaceae* family [44, 45]. Various researchers also came to this conclusion [46, 47]. According to Ibrahim et al. [48], just 2.6% of the Gram-negative isolates tested were resistant to gentamycin at concentrations up to 10 g, while 2.1% displayed resistance at concentrations up to 256 g. This makes gentamycin the most potent antibiotic against Gram-negative isolates. The results of the current analysis support Tayyab et al. earlier report that multidrug resistance is highly recurrent [49]. Usha et al. [50] provided additional details on comparable discoveries. More than other microorganisms, the majority of Gram-negative bacterial isolates showed elevated resistance, according to Esposito and Leone [51]. An overall drug similarity score is determined by molecular characteristics and drug likelihood. Molecules that meet Lipinski's “rule of five,” molecular weight >500, log *P* > 5, hydrogen bond acceptors >10, and hydrogen bond donors >5, show poor absorption or penetration rate. Lipinski's “rule of five” states that the chemical might make a human orally active medication. The distribution of the medication's overall drug-likeliness score is tilted to the right and falls between 0.8 and 1.2 [52, 53]. The primary factor that led to the termination of drug candidate development was drug toxicity [54]. Due to a lack of ADME/Tox throughout development, more than 50% of medicines were unsuccessful. ADME reduces the likelihood of failure during the early stages of in vitro, which are still time-andresource-intensive. In order to quickly calculate drug-likeness and ADME/Tox data, a new web-based function called Pre ADMET has been developed [55]. Several in vitro methods have been used to assess the intestinal absorption of medication candidates throughout the drug selection process. It has been suggested that the Caco2-cell model and the MDCK (Madin-Darby canine kidney) cell model are reliable in vitro models for the assessment of oral medication absorption. This module offers prediction models for the in vitro Caco2-cell [56] and MDCK cell [57] assays for absorption. Additionally, the human intestine absorption model and the in silico skin permeability model can be used to find prospective drugs for transdermal and oral delivery. Blood-brain barrier (BBB) penetration can provide data on therapeutic medication distribution, plasma protein binding (PPB) model efficacy, and disposition in the central nervous system. Both noninfectious and unstoppable bacteria produce extracellular proteolytic enzymes. When produced by irresistible bacteria, these chemicals can be fatal to the host and are essential to their life cycles [58]. All bacterial isolates produce amylase and protease, but only a few strains of Gram-negative microorganisms can make lipase proteins, according to Nailah et al. [59]. The *P. vulgaris* isolates used in this study produce the enzymatic activities of amylase, beta-lactamase, protease, lipase, gelatinase, and urease. The majority of the combined isolates, 98%, 84%, and 80%, showed individual activity for urease, gelatinase, amylase, and lipase. Some other experts made arrangements for our study [60, 61]. The proliferation of antimicrobial and heavy metal resistance properties depends mostly on plasmids, which are extrachromosomal DNA fragments capable of copying just the genome. Plasmids and conjugative transposons function as crucial intermediaries in conjugation, a process that has a big impact on bacterial development and motility. The majority of horizontal gene exchanges facilitated by plasmids are capable of obtaining antimicrobial resistance (AMR) genes [62]. Nine *P. vulgaris* isolates (PR 7, PR 8, PR 27, PR 9, PR 27, PR 30, PR 48, PR 43, and PR 46) included just a single plasmid from all the sites investigated, but one isolate (PR 47) in the current investigation contained multiple plasmids. A few additional researchers back up our work [63]. Plasmids have expressed concern over the acquisition of defence against many anti-infections and heavy metals [64]. It is well accepted that one key mechanism in the selective adaptation of bacteria to changes in the local environment is the level interchange of genes within and among the bacterial population. Conjugative exchange is the most effective mechanism for even gene transfer, and it is for this reason that it is thought to be one of the main motivations for the proliferation of microbes with multiple anti-infection and metal defences. In the current study, numerous donor strains of *P. vulgaris* isolates that were classified as Gram-negative bacteria beneficiaries showed antimicrobial metal resistance. *E. coli* K-12 and *P. vulgaris* isolates in supplement stock are contrasted with wastewater. Our study's findings are consistent with those of earlier studies, which demonstrate that Gram-negative bacteria spread resistance more frequently in hospitals [65]. This finding suggested that conjugative plasmids, as opposed to chromosomes, which were easily transferred to *E. coli* K-12, were responsible for a significant portion of the multiresistance. Conjugation was carried out in a development environment with a nutrient medium and wastewater. Under nutritional medium and hospital wastewater, conjugation sets demonstrated a two-way exchange of resistance transfer. By exchanging genetic material at a high enough level, antimicrobial resistance can spread. Different conjugation, transformation, or transduction methods may be used to exchange antimicrobial resistance genes. Public health is at risk from the significant growth in antimicrobial resistance bacterial contaminations demonstrated by *P. vulgaris*, including resistance transfer in organisms, particularly given the rise in resistance and microbial population in hospital wastewater. In this study, the distribution of antimicrobial drug resistance against *P. vulgaris* in hospital wastewater was examined without distinguishing between transferable and nontransferable resistance. In instances when population management is not professionally carried out, it is recommended that bacteria linked to healing centre disorders be able to live for extended periods of time [66, 67].

## 5. Conclusion

In conclusion, hospital wastewater is harmful to the environment. The extent to which hospital wastewater contributed to the *P. vulgaris* resistance which was developing in North Indian hospitals was made clear by the current analysis. We looked at extracellular enzyme activity, including protease, amylase, lipase, beta-lactamase, catalase, gelatinase, and urease, in metal-tolerant and antibiotic-resistant *P. vulgaris* isolates. According to the study, the majority of heavy metal and antibiotic-resistant *P. vulgaris* isolates contain plasmids, which are both prevalent and important in the development and transfer of resistance. The present work raises awareness about the resistance among these species, which is of utmost relevance since these species are a potential source of resistance genes that may be passed on to other bacterial diseases. All of the medications used in this experiment were further validated by using Lipinski's rule of five and ADME-Tox, which was discovered to fit within the range of drug-like properties. This opens the door for further investigation into the prevalence of antibiotic resistance in *P. vulgaris* infections found in hospital settings.

## Figures and Tables

**Table 1:** Correlation coefficient (*R*), slope, and residual mean square (RMS) values were obtained for the exponential decay model for hospital wastewater at different concentrations of metals.

Sites	Metals	*R*	Slope	RMS
1	Hg	0.377	−805.7	0.315765696
	Cd	0.672	−13430	0.315587972
	Co	0.693	−13636	0.304682265
	Ni	0.896	−13547	0.309583244
	Zn	0.682	−13470	0.399291110
	Cr	0.610	−14185	0.284863178
	Cu	0.883	−14251	0.038969882

2	Hg	0.383	−242.8	0.312361700
	Cd	0.548	−14968	0.303305862
	Co	0.846	−93786	0.406099135
	Ni	0.884	−25693	0.034904556
	Zn	0.621	−17268	0.210423823
	Cr	0.534	−15614	0.274040907
	Cu	0.916	−14918	0.016719939

3	Hg	0.377	−628.5	0.316530857
	Cd	0.759	−14671	0.405153449
	Co	0.658	−24644	0.103931115
	Ni	0.692	−14510	0.376696231
	Zn	0.606	−74177	0.392370443
	Cr	0.410	−14795	0.376008990
	Cu	0.594	−14468	0.432005013

4	Hg	0.376	−248.5	0.316005435
	Cd	0.686	−88086	0.395312693
	Co	0.674	−88132	0.419002505
	Ni	0.934	−89289	0.042191565
	Zn	0.743	−90571	0.400534444
	Cr	0.420	−90761	0.365269321
	Cu	0.621	−87379	0.436048750

**Table 2:** Correlation coefficient (*R*), slope, and residual mean square (RMS) values were obtained for the exponential decay model for hospital wastewater at different concentrations of antibiotics.

Sites	Antibiotics	*R*	Slope	RMS
1	E	0.770	−17905	0.400644778
	TET	0.654	−17868	0.404152601
	P	0.645	−17870	0.398565533
	AMP	0.635	−17861	0.399742227
	AMX	0.600	−17858	0.402387831

2	E	0.705	−18937	0.364518694
	TET	0.548	−18706	0.382043281
	P	0.633	−18503	0.3828429
	AMP	0.915	−19423	0.032093116
	AMX	0.693	−18649	0.385588529

3	E	0.833	−19318	0.397037153
	TET	0.632	−19261	0.355218298
	P	0.732	−19297	0.394136368
	AMP	0.752	−17861	0.391330446
	AMX	0.715	−19270	0.399837324

4	E	0.642	−11628	0.397655813
	TET	0.682	−11645	0.314915386
	P	0.832	−11740	0.390125225
	AMP	0.864	−11685	0.039767905
	AMX	0.729	−11642	0.40160886

**Table 3:** Metal resistance patterns in *P. vulgaris* (*n* = 50) from the entire sampling site.

No. of metals	Resistance pattern	Isolates	Percentage (%)	MMR index
2	Ni, Zn	1	6	0.09
	Cr, Ni	2		
3	Cr, Ni, Zn	1	2	0.42
4	Hg, Cr, Ni, Zn	1	24	0.04
	Cr, Cu, Ni, Zn	8		
	Cr, Cu, Cd, Zn	1		
	Cu, Ni, Cd, Zn	1		
	Cr, Ni, Cd, Zn	1		
5	Co, Cr, Cu, Ni, Zn	6	24	0.05
	Co. Cr, Cu, Ni, Cd	1		
	Hg, Co, Cr, Cu, Ni,	1		
	Hg, Co, Cr, Ni, Zn	2		
	Cr, Cu, Ni, Cd, Zn	2		
6	Co, Cr, Cu, Ni, Cd, Zn	9	40	0.04
	Hg, Co, Cr, Cu, Ni, Zn	5		
	Hg, Cr, Cu, Ni, Cd, Zn	6		
7	Hg, Co, Cr, Cu, Ni, Cd, Zn	2	4	0.5

Cr = chromium, Cd = cadmium, Co = cobalt, Hg = mercury, Cu = copper, Zn = zinc, Ni = nickel.

**Table 4:** Percentage of resistance against antibiotic in *P. vulgaris* isolated from entire sampling sites.

Antibiotics	Concentration (*μ*g/disc)	No. of proteus resistant (*n* = 50)	Percentage (%)
Amoxicillin	30	30 (60%)	60
Ofloxacin	5	3 (6%)	6
Erythromycin	15	10 (20%)	20
Gentamycin	50	5 (10%)	10
Methicillin	5	50 (100%)	100
Nalidixic acid	30	19 (38%)	38
Neomycin	30	5 (10%)	10
Penicillin	10	43 (86%)	86
Nitrofurazone	100	5 (10%)	10
Chloramphenicol	30	7 (14%)	14
Tetracycline	10	6 (12%)	12
Ciprofloxacin	5	8 (16%)	16
Kanamycin	30	8 (16%)	16
Polymixin B	300	23 (46%)	46
Ampicillin	10	39 (78%)	78

**Table 5:** Antibiotic resistance patterns in *P. vulgaris* (*n* = 50) from the hospital wastewater.

No. of antibiotics	No. of resistant isolates (%)	Resistance pattern	Percentages	MAR index
1	1(2)	M	2	
2	1 (2)	M, AMP	12	0.02
	3 (6)	M, P		
	1 (2)	M, NA		
	1 (2)	AMP, K		
3	1 (2)	NA, AMX, TET	12	0.03
	1 (2)	M, AMX, AMP		
	1 (2)	M, AMOX, P		
	3 (6)	M, AMP, P		
4	3 (6)	M, AMP, P, PBY	18	0.02
	3 (6)	M, AMOX, AMP, P		
	1 (2)	M, AMP, N, PB		
	1 (2)	M, AMP, P, E		
5	1 (2)	M, NA, AMP, P, E	12	0.05
	1 (2)	M, NA, AMP, P, PB		
	1 (2)	M, AMX, AMP, P, PB		
	1 (2)	M, AMX, K, P, PB		
	1 (2)	M, AMX, AMP, P, CIP		
	1 (2)	M, NA, AMX, AMP, P		
6	2 (4)	M, NA, AMX, AMP, P, PB	16	0.05
	1 (2)	M, AMX, AMP, P, N, PB		
	1 (2)	M, NA, AMX, AMP, P, CIP		
	1 (2)	M, NA, P, NR, PB, CIP		
	1 (2)	M, AMP, P, OF, GENT, PB		
	1 (2)	M, AMX, AMP, P, TET, PB		
	1 (2)	M, NA, AMX, AMP, P, PB, CIP		
7	2 (4)	M, NA, AMX, AMP, P, PB, CIP	16	0.05
	1 (2)	M, AMX, AMP, P, NR, PB, E		
	1 (2)	M, NA, K, AMP, P, N, PB		
	1 (2)	M, NA, AMX, K, AMP, P, TET		
	2 (4)	M, NA, AMX, AMP, P, CHL, E		
	1 (2)	M, NA, AMX, AMP, P, PB, E		
9	1 (2)	M, AMOX, K, AMP, P, TET, NR, CHL, E	6	0.2
	1 (2)	M, NA, AMX, K, AMP, P, GENT, N, TET, E		
	1 (2)	M, NA, AMX, AMP, P, NR, PB, CHL, E		
11	1 (2)	M, NA, AMX, K, AMP, P, GENT, CHL, PB, CIP, NR	2	0.7
12	1 (2)	M, AMX, K, AMP, P, GENT, N, TET, CHL, PB, CIP, E	4	0.4
	1 (2)	M, NA, AMX, AMP, P, OF, GENT, N, CHL, CIP,		

AMX = amoxicillin, OF = ofloxacin, E = erythromycin, GEN = gentamycin, M = methicillin, NA = nalidixic acid, N = neomycin, P = penicillin, NR = nitrofurazone, CHL = chloramphenicol, TET = tetracycline, CIP= ciprofloxacin, K = kanamycin, PB = polymixin B, AMP = ampicillin.

**Table 6:** Extracellular enzymatic activities in various species of *P. vulgaris* from the hospital wastewater.

Enzyme	Percentage (%)
Beta-lactamase	56
Lipases	80
Proteases	68
Amylases	80
Catalase	74
Gelatinase	84
Urease	98

**Table 7:** Incidence of resistance transfer among the metal-tolerant *P. vulgaris* isolates from hospital wastewater.

Donor strains	Pattern of donor strains	Recipient strains	Pattern of recipient strains	Frequency of transfer in nutrient broth	Frequency of transfer in wastewater	Pattern of transconjugants	Markers transferred
PR 7	Hg, Co, Cu, Ni, Cd, Zn	PR8	Cu, Ni, Cd,	3.4 × 10^−1^	2.4 × 10^−1^	Co, Cr, Cu, Ni	Zn, Cr
PR 8	Cu, Ni, Cd	PR9	Hg, Co, Ni, Cd,	1.0 × 10^−2^	0	Co, Cr, Cu, Ni, Zn	Cu
PR 27	Hg, Co, Cr, Cu, Ni, Cd, Zn	PR46	Cr, Cu, Ni, Cd, Zn	1.5 × 10^−1^	2.8 × 10^−1^	Cr, Cu, Ni, Cd, Zn	Cr, Co
PR 30	Co, Cr, Cu, Ni, Cd	PR4	Cr, Cu, Ni, Zn	1.2 × 10^−1^	0	Hg, Co, Cr, Cu, Ni, Cd, Zn	Co, Cd
PR 38	Co, Cr, Cu, Ni, Zn	PR17	Cr, Ni	8.0 × 10^−2^	3.1 × 10^−1^	Co, Cr, Cu, Ni, Zn	Zn, Cu
PR 43	Hg, Co, Cr, Cu, Ni, Cd, Zn	PR38	Hg, Co, Cr, Cu, Ni, Zn	1.6 × 10^−1^	1.8 × 10^−1^	Co, Cr, Cu, Ni, Cd, Zn	Cd
PR 47	Co, Cr, Cu, Ni, Cd, Zn	PR25	Cr, Zn	0.0	5.0 × 10^−2^	Co, Cr, Cu, Ni, Zn	Co, Ni

Co = cobalt, Cr = chromium, Hg = mercury, Ni = nickel, Zn = zinc, Cu = copper, Cd = cadmium.

**Table 8:** Transfer of antibiotic resistance in resistant *P. vulgaris* isolates from hospital effluent.

Donor strains	Pattern of donor strains	Recipient strains	Pattern of recipient strains	Frequency of transfer in nutrient broth	Frequency of transfer in wastewater	Pattern of transconjugants	Markers transferred
PR 7	M, NA, AMX	PR8	M, K, N, PB	2.0 × 10^−2^	2.1 × 10^−1^	M, NA, AMX, AMP, P	AMX, AMP
PR 8	M, NA, AMX, AMP, P	PR9	M, P, K, NR, PB	2.8 × 10^−1^	4.4 × 10^−1^	M, NA, AMX, AMP, P	AMX, AMP
PR 27	M, NA, AMX, AMP, P	PR46	M, P, PB	1.0 × 10^−2^	0.0	M, NA, AMX, AMP, P, PB	AMX, AMP
PR 30	M, AMX, AMP, P, K, OF, GENT, N, CHL, PB, E, CIP TET	PR4	M, P, AMP	3.0 × 10^−1^	0.0	M, AMX, AMP, P, PB, E	AMX, ERY
PR 38	M, AMX, AMP, K, P, GENT, N, TET, CHL, PB, E, CIP	PR17	K, AMP, P	3.1 × 10^−1^	0.0	M, NA, AMX, AMP, P, E	AMX
PR 43	M, NA, AMX, AMP, P, N	PR38	M	5.0 × 10^−2^	5.0 × 10^−2^	M, NA, AMX, K, AMP, P, OF, GENT, TET, CIP, E	AMX, AMP, TET
PR 47	M, AMX, AMP, K, P, GENT, N, TET, CHL	PR25	M, NA, AMX, AMP, P, NR, CHL, PB, E	1.0 × 10^2^	2.9 × 10^−1^	M, AMX, AMP, K, P, N, TET, NR, CHL	TET

AMX = amoxicillin, OF = ofloxacin, E = erythromycin, GENT = gentamycin, M = methicillin, NA = nalidixic acid, N = neomycin, P = penicillin, NR = nitrofurazone, CHL = chloramphenicol, TET = tetracycline, CIP = ciprofloxacin, K = kanamycin, PB = polymixin B, AMP = ampicillin.

**Table 9:** Incidence of resistance transfer among the antibiotic/heavy metal-resistant *P. vulgaris* isolates to *E. coli* K-12 from hospital wastewater.

*Donor Strains*	Pattern of donor strains	Recipient strains	Frequency of transfer in nutrient broth	Frequency of transfer in wastewater	Pattern of transconjugants	Markers transferred
PR 17	Hg, Co, Cr, Cu, Ni, Cd, Zn	*E.coli* K-12	3.3 × 10^−1^	1.1 × 10^−1^	Cr, Cu, Ni, Zn	Ni, Cr
PR 17	M, NA, AMX, AMP, P	*E.coli* K-12	2.9 × 10^−1^	0	M, AMX, AMP, K, P	AMX, AMP

**Table 10:** Drug-likeness properties of the different drugs used in this study.

S. no	Compounds	Molecular weight	No. of HBA	No. of HBD	MolLogP	Drug-likeness model score:
1.	Amoxicillin	365.10	7	5	0.05	1.39
2.	Chloramphenicol	323.02	5	4	0.17	0.93
3.	Ampicillin	349.11	6	4	0.31	1.11
4.	Erythromycin	733.46	14	5	1.38	1.48
5.	Gentamycin	477.32	11	12	−5.08	0.85
6.	Methicillin	380.10	7	2	2.41	0.29
7.	Nalidixic acid	232.08	4	1	1.23	0.82
8.	Neomycin	614.31	19	19	−12.21	0.97
9.	Penicillin	334.10	5	2	2.55	0.80
10.	Ciprofloxacin	331.13	4	2	1.29	0.93
11.	Kanamycin	484.24	15	15	−9.45	0.95
12.	Nitrofurazone	114.03	3	1	−0.76	−1.23
13.	Ofloxacin	361.14	5	1	1.21	1.20
14.	Polymixin B	1202.75	18	23	−7.48	−1.10
15.	Tetracycline	444.15	9	7	−1.17	1.43

No. of HBA: no. of hydrogen bond acceptor. No. of HBD: no. of hydrogen bond donor.

**Table 11:** ADME/Tox prediction of the different used drugs.

S. no.	Compounds	CaCO2	HIA	MDCK	PPB	SP	Toxicity (*M*/*C*_*m*,_*C*_*r*_)
1.	Amoxicillin	0.3483	62.936	0.3985	23.3530	−5.149	+/−, −
2.	Chloramphenicol	17.5665	83.2759	1.3436	65.9271	−4.053	+/−,−
3.	Erythromycin	23.3211	52.9870	0.04341	36.0565	−2.859	−/−, −
4.	Gentamycin	4.4695	5.2795	0.46653	46.930	−5.184	+/−, −
5.	Methicillin	14.9867	87.322	2.4672	56.0535	−4.704	+/−, −
6.	Nalidixic acid	0.276663	96.2385	30.345	76.8196	−3.888	+/+, −
7.	Neomycin	1.7782	0	0.5092	43.566	−5.305	+/−, −
8.	Penicillin	11.859	92.7501	3.13642	82.5857	−4.546	-/-, -
9.	Ciprofloxacin	21.280	96.270	10.302	31.053	−4.597	+/−, −
10.	Kanamycin	2.015	0.13581	0.5170	42.225	−5.279	+/−, −
11.	Nitrofurazole	20.107	74.332	37.430	0	−2.944	+/−, −
12.	Ofloxacin	25.943	98.3543	13.764	28.788	−4.629	+/−, −
13.	Polymyxin B	20.4008	0	0.0434	44.899	−2.290	+/+, −
14.	Tetracycline	19.274	35.2166	0.50796	33.2060	−4.672	−/−, −
15.	Ampicillin	0.6307	81.4784	0.93758	36.154	−5.035	+/−, −

CaCO2 = Caco2 cell permeability, HIA = human intestinal absorption, MDCK = Madin-Darby canine kidney, PPB = plasma protein binding, SP = skin permeability.

## Data Availability

Data presented in this study are available upon request.
